# Diabetes screening among women with Polycystic Ovary Syndrome: A descriptive study of commercial claims, 2011–2019

**DOI:** 10.21203/rs.3.rs-4214680/v1

**Published:** 2024-04-15

**Authors:** Jacklyn Vollmer, Mary E. Lacy, W. Jay Christian

**Affiliations:** University of Kentucky; University of Kentucky; University of Kentucky

**Keywords:** PCOS, diabetes, screening, women, hormonal disorder

## Abstract

**Background:**

Polycystic ovary syndrome (PCOS) is a hormonal disorder that affects 6–12% of United States women of reproductive age. Women with PCOS are at an increased risk of developing type 2 diabetes and fall into high-risk groups according to the American College of Obstetricians and Gynecologists (ACOG) screening guidelines. Guidelines further indicate that an oral glucose tolerance test (OGTT) should be used for diabetes screening in women with PCOS instead of an A1C or fasting plasma glucose test. The purpose of this study is two-fold: 1) to estimate rates of diabetes screening among a nationwide sample of commercially insured women with PCOS and 2) to report the percentage of women screened using each test (OGTT, A1C, fasting plasma glucose) among those who were screened.

**Methods:**

We used the MarketScan Commercial Claims database (2011–2019) to identify a sample of women aged 18–64 years with PCOS who were free from diabetes at baseline and had ≥ 5 years of continuous enrollment. PCOS was ascertained using International Classification of Disease diagnosis codes (ICD-9: 256.4; ICD-10: E28.2). Diabetes screening was ascertained using Current Procedural Terminology (CPT) codes (A1C: 83036, 83037; Fasting blood sugar: 82947; OGTT: 82950). Diabetes screening rates were calculated for the overall study sample as well as across subgroups defined by age, overweight/obesity, hypertension, hypercholesterolemia, and vascular disease.

**Results:**

In our sample of 191,110 commercially insured women with PCOS, 73.40% were screened at least once for diabetes during a five-year period. Among the women screened, 19.24% were screened using the Androgen Excess Society (AES)-recommended OGTT, 61.58% were screened using A1C, and 23.37% were screened using fasting blood sugar.

**Conclusions:**

Almost 75% of women with PCOS comply with the ACOG screening guidelines for diabetes. However, while the OGTT is recommended as the preferred screening tool for women with PCOS, it was less commonly used than A1C and fasting blood sugar tests.

## BACKGROUND

Polycystic ovary syndrome (PCOS) is a hormonal disorder characterized by the ovaries producing an abnormal amount of androgen (male sex hormones). PCOS impacts 6–12% of US women of reproductive age and causes symptoms such as irregular periods, excess body hair, weight gain, acne, and infertility.^1,^ In addition to these symptoms, PCOS also increases the risk of long-term complications like heart disease, endometrial and other cancers, and diabetes.^1,,^ By the age of 30 years, ~ 25–30% of women with PCOS will have impaired glucose tolerance.^2,^ Because of this increased risk of diabetes, the American College of Obstetricians and Gynecologists (ACOG) and the American Diabetes Association (ADA) recommend that all women with PCOS should be screened for diabetes at PCOS diagnosis and, upon normal results, screening should be repeated at 3–5-year intervals.^2,5^

Screening for diabetes can be conducted using measures of hemoglobin A1C, fasting glucose, random glucose, or the oral glucose tolerance test (OGTT). However, an expert panel appointed by the Androgen Excess Society (AES) recommends that women with PCOS be screened for diabetes with an OGTT rather than other screening measures. This recommendation is based on prior studies showing that, in women with PCOS, fasting glucose failed to recognize glucose intolerance in 58% of cases screened positive by OGTT.^3,7^

Despite the importance of screening in this population of women at high risk for developing diabetes, few studies have estimated compliance with screening guidelines. One study identified patients with PCOS (n = 547) at an academic medical center in 2006 and 2011 and, in both time periods, reported that ≤ 25% completed laboratory screening tests for diabetes. Another study reported that less than 57% of gynecologists and 71% of reproductive endocrinologists screened for diabetes in women newly diagnosed with PCOS.^4^ Even fewer of these gynecologists (49%) and reproductive endocrinologists (53%) performed re-screening for diabetes following the initial screen at PCOS diagnosis.^9^ Another study reported similar low rates, with ≤ 25% of PCOS patients undergoing any screening.^12^ Understanding current compliance with clinical guidelines for diabetes screening in this high-risk population is important to inform next steps. In this study, we estimated rates of diabetes screening among a nationwide sample of commercially insured women with PCOS in the U.S., and among those who were screened, determined the percentage of women screened using each type of test (OGTT, A1C, fasting plasma glucose). We also compared how screening rates differ among women with PCOS alone versus PCOS plus additional diabetes risk factors (age, overweight or obesity, hypertension, hypercholesterolemia, and vascular disease).

## METHODS

### Study Population and Data

We conducted a cross-sectional, descriptive study using the Merative MarketScan database to examine rates of diabetes screening among women with PCOS. Merative MarketScan data is a claims database that includes data on approximately 3 million enrollees annually who have employer-sponsored insurance. For the current study, we identified all women aged 18–64 years who were free from documented diabetes (no indication of diabetes-related diagnoses: ICD-9: 249–250; ICD-10: E08-E13) at the time of their first documented PCOS diagnosis (ICD-9 256.4 or ICD-10 E28.2) during the study period (1/1/2011 to 12/31/2019). This resulted in an initial sample of 301,902 women. We then restricted the population to women with a maximum of five years of continuous enrollment in the MarketScan database, starting with first PCOS diagnosis, to create a population of women with PCOS who were free from diabetes and, based on ACOG and ADA guidelines, should have been screened for diabetes at least once during the study period, resulting in a final analytic sample of n = 191,110 women.^2,5^

### Outcome Ascertainment

The primary outcome was diabetes screening ascertained via Current Procedural Terminology (CPT) codes (83036, 83037, 82947, 82950). CPT codes were extracted starting on the date of first PCOS diagnosis during the study period up to a maximum of five years following PCOS diagnosis. As a secondary analysis, among those who were screened for diabetes, we examined what type of screening test was performed using CPT codes (A1C: 83036, 83037; fasting blood sugar: 82947; OGTT: 82950).

### Covariates

In addition to PCOS, we examined presence/absence of select diabetes risk factors that may influence compliance with screening guidelines, including age; overweight or obesity (ICD-9: 278.0, ICD-10: E66); hypertension (ICD-9: 401.x, ICD-10: I10); hypercholesterolemia (ICD-9: 272, ICD-10: E78); and vascular disease (myocardial disease, congenital heart disease, stroke (ICD-9: 410, 430, 431, 433.x1, 434.x1, 435, 436, 362.3, 414.9, ICD-10: I21.09, I21.19, I21.11, I21.29, I21.4, I21.9, I60, I61, I63, I64, H34.1, G45.1, I25)).

#### Statistical Analysis

We tabulated the distribution of demographic and clinical covariates of the overall sample. Next, we examined the prevalence of diabetes screening overall, by test type and across categories of number of additional diabetes risk factors among women with PCOS.

In addition to PCOS, we categorized participants based on presence/absence of select diabetes risk factors (overweight/obesity, hypertension, hypercholesterolemia, vascular disease, age ≥ 45 years). We examined screening patterns across categories of a summary score of the number of additional risk factors present in addition to PCOS: 0 additional risk factors, 1 additional risk factor, 2 additional risk factors, 3 additional risk factors, and ≥ 4 additional risk factors. We also calculated the time from a patient’s first PCOS diagnosis in the study period to their first diabetes screening test performed in the study period. Finally, we examined the association between diabetes screening and presence/absence of individual risk factors using logistic regression models adjusted for age and time in the study. Statistical significance was set at p ≤ 0.05. Data were analyzed using SAS Studio Release 3.8 (Enterprise Edition) (SAS Institute Inc., Cary, NC, USA. 2012–2018).

## RESULTS

Selected demographic, clinical, and social characteristics of study participants are displayed in Table 1. Study participants had a mean age of 30.82 years old (± 9.13) at PCOS diagnosis, with 6.91% of the population aged 45 years or older. The average time enrolled for our study participants was 4.31 (± 0.71) years. The most common risk factor for our sample was overweight/obesity (27.59%), followed by hypercholesterolemia (18.06%), hypertension (14.82%), and vascular disease (0.72%).

**Figure 1:**
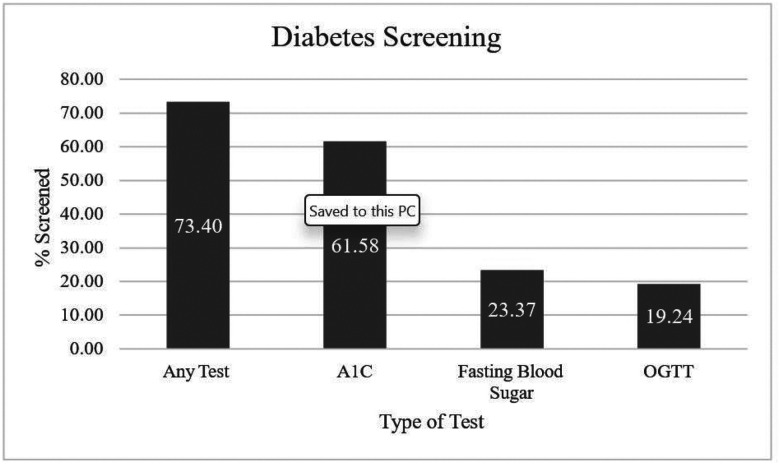
Prevalence of diabetes screening among women aged 18–64 with PCOS, 2011–2019 Prevalence of diabetes screening, overall and by type of screening test, among women aged 18–64 with PCOS from the Merative MarketScan Commercial Claims and Encounters Database, 2011–2019; Abbreviations: A1c: hemoglobin A1c; OGTT: oral glucose tolerance test

The majority of women in our sample (73.40%) were screened at least once for diabetes during the study period (Fig. 1). The most commonly used diabetes test was A1C, with 61.58% of women in our sample screened by A1C. Fasting plasma glucose (23.37%) was the second most common test, followed by OGTT (19.24%). Some individuals 35.37%) were screened by two or more of these tests.

Table 2 displays the prevalence of diabetes screening overall and average time to screening from diagnosis by test type, and across categories of additional diabetes risk factors. The percentage of women ever screened increases as the number of risk factors increases. Women with four or more risk factors in addition to PCOS had the highest prevalence of diabetes screening (86.07%) while those with zero additional risk factors had the lowest (68.40%). Across all risk factor groups, A1C was the most commonly used screening test and OGTT was the least commonly used. The average time to screening from PCOS diagnosis increased as the number of risk factors increased. Women with one risk factor had a longer time to screening (339.22 days) than women with 4 or more risk factors (243.52 days).

Finally, in logistic regression models, we examined odds of diabetes screening based on presence/absence of additional diabetes risk factors (Table 3). The highest screening rates were found in women with hypercholesterolemia (80.93%), followed by women who were overweight/obese (79.84%), women with hypertension (79.15%), and those with vascular disease (77.30%). In unadjusted and adjusted models, presence of additional risk factors increased odds of screening. In adjusted models, women with PCOS who were also overweight or obese were 1.66 (CI:1.62, 1.70) times more likely to be screened for diabetes than women with PCOS who were not overweight/obese. Presence of hypercholesterolemia (OR = 1.68; CI: 1.63,1.73), hypertension (OR = 1.44; CI: 1.40, 1.49), and vascular disease 1.23 (1.08, 1.39) were also associated with greater odds of screening as compared to women with PCOS but without the additional risk factor. Women with PCOS who were ≥ 45 years old were less likely to be screened than those with PCOS who were < 45 years old (OR = 0.96; 95% CI: 0.92, 0.99).

## DISCUSSION

In a sample of 191,110 women from the Merative MarketScan database, 73.40% of women with PCOS were screened at least once for diabetes during our 5-year study period. Out of women who were screened, however, only 19.2% were screened using the AES-recommended OGTT;^7^ A1C and fasting glucose, which are less reliable tests for women with PCOS, were nonetheless more commonly used to screen for diabetes. Screening for diabetes in women with PCOS is crucial as these women have a higher risk of developing diabetes.^2,3,7,^ In a retrospective analysis, the crude incidence rate of diabetes was 14.25/1000 person-years in women with PCOS compared to 3.45 in those without PCOS. Additionally, a similar study found that PCOS in women was associated with a twofold higher odds of incident diabetes.

Overall, a relatively high percentage of the women in our study were screened at least once for diabetes. Though literature is limited, two prior studies reported lower rates of diabetes screening among women with PCOS. One study of PCOS patients identified at an academic medical center in 2006 and 2011 and, in both time periods, reported that ≤ 25% of PCOS patients completed laboratory screening tests for diabetes.^8^ Another study surveyed obstetrician-gynecologists to better understand their routine screening practices for patients with PCOS; this study reported that approximately half of respondents (53.2%) stated that they would order diabetes screening tests at least every 5 years on their patients with PCOS following initial results being normal (53.2% stated they would order A1c testing at least every five years; 53.8% fasting glucose testing; 47.1% OGTT).^9^ Many factors may contribute to the high “screened at least once” rate in this study. First, we are evaluating screening over an extended time frame (up to 5 years). The most relaxed interpretation of current guidelines would support screening at least once in a 5-year interval.^2,5^ Second, this is a population of commercially insured individuals who may have better access to preventive care than the general US population. Third, in our study population, roughly half of women had additional diabetes-related risk factors beyond PCOS. Screening recommendations for these additional risk factors may have also prompted women in the study to be screened for diabetes. Indeed, rates of diabetes screening increased with each additional diabetes-related risk factor, ranging from 68% in those with no additional risk factors to 86% in those with ≥ 4 risk factors.

We found that the A1C test was the test that was most frequently used to screen for diabetes in this sample. A1C testing offers a number of advantages over glucose-based tests. One of the main advantages of using an A1C test is that fasting is not required and factors such as diet, stress, and exercise do not affect testing. A1C testing has also been found to be superior to measure chronic hyperglycemia as opposed to two assessments of fasting blood sugar or OGTT tests.^13^ In addition, the stability of A1C tests from whole blood samples is greater than plasma glucose tests.^13^ Despite these advantages, however, prior guidelines from an expert panel appointed by the Androgen Excess Society (AES) recommends that women with PCOS be screened for diabetes with OGTT rather than other screening measures as it was more accurate at identifying glucose intolerance.^3,7^ Our findings reveal relatively high overall rates of diabetes screening in this population but highlight a gap in compliance with guideline-recommended OGTT use.

Study strengths include a population representative of the entire US and use of diagnostic and CPT code data rather than self-reported data on diabetes screening. Despite these strengths, there are a number of limitations. One limitation of this study was the inability to obtain information on additional diabetes risk factors such as race and ethnicity, family history of diabetes, and physical activity levels. These additional risk factors may have influenced screening frequency and type of test, but these variables were not available through the Merative MarketScan database. We were also unable to differentiate between overweight and obese, which also could have impacted screening type and frequency. In addition, our ascertainment of PCOS was based on diagnosis codes alone. The data in the Merative MarketScan database are claims-based data and, as such, were collected for reasons other than research. It is possible that our study population included some women with a PCOS diagnosis that was a “rule-out” diagnosis and, likewise, it is possible that some women who truly had PCOS were not captured if they did not have a diagnosis code indicative of PCOS during the study period. This study also has a limitation on its generalizability as it only includes data from commercially insured individuals. Future studies should include individuals with publicly funded insurance such as Medicare or Medicaid to fully understand the patterns in these different groups. Finally, in 2022, the ADA broadened diabetes screening guidelines to include all adults aged 35 and older regardless of presence of additional risk factors, including PCOS; previous guidelines recommended screening starting at age 45. As a result, this analysis does not conform to the new guidelines and, as such, does not examine screening patterns in those 35 and older.

### Conclusion

In this study of nearly 200,000 women with PCOS, we found that nearly 75% of women were screened for diabetes at least once in a five-year period. We also found that, while the OGTT is the recommended screening test for this population, OGTT was the least commonly used screening test. To further explore this area and to better understand diabetes testing within women with PCOS, future research might explore provider recommendations and interactions with their patients. Additional research should also look into the frequency and type of tests and their relationship with PCOS in combination with all additional risk factors for diabetes that were not available within the Merative MarketScan database, such as race/ethnicity, family history, and physical activity levels.

## Figures and Tables

**Table 1:** Distribution of baseline characteristics of women aged 18–64 with PCOS, Merative MarketScan claims database (2011–2019)

	N = 191,110
Demographics	
Age at first PCOS diagnosis (years), mean (SD)	30.82 (± 9.13)
Average time in study (years), mean (SD)	4.31 (± 0.71)
Region, n (%)	
North Central	34485 (18.04)
Northeast	41195 (21.56)
South	86809 (45.05)
West	27268 (14.27)
Unknown	1773 (0.93)
Risk Factors, n (%)	
Age ≥45*	13198 (6.91)
Overweight/Obese*	52736 (27.59)
Hypertension*	28321 (14.82)
Hypercholesterolemia*	34513 (18.06)
Vascular Disease*	1379 (0.72)

PCOS, Polycystic Ovary Syndrome; SD, Standard Deviation; ICD, International Classification of Disease

* Risk factors for diabetes ascertained via ICD9 and ICD10 codes.

**Table 2:** Prevalence of diabetes screening by additional diabetes risk factors among women with PCOS

		Screened for diabetes		Type of Test		
Number of diabetes risk factors	Total, n (%)	Yes (%)	Time to first screening (days), mean (SD)	A1C (%)	Fasting glucose (%)	OGTT (%)
Overall		73.40	361.61(± 430.47)	61.58	23.37	19.24
0	99217 (51.92)	68.40	404.85(± 449.89)	52.60	22.88	22.43
1	56488 (29.56)	77.01	339.22(± 419.04)	67.91	24.40	17.83
2	24607 (12.88)	80.77	302.70(± 393.49)	75.09	23.67	13.95
3	8688 (4.55)	83.11	281.98(± 378.74)	79.30	21.88	9.86
≥ 4	2110 (1.10)	86.07	243.52(± 346.81)	83.51	21.18	7.20

PCOS, Polycystic Ovary Syndrome; A1C, Hemoglobin A1C; OGTT, Oral Glucose Tolerance Test; SD, Standard Deviation

* Risk factors are in addition to PCOS and include age ≥ 45, overweight/obese, hypertension, hypercholesterolemia, vascular disease

**Table 3:** Association between diabetes risk factors and odds of diabetes screening among women with PCOS

	Odds of diabetes screening		
	Screened n (%)	Odds Ratio (95% CI)	Adjusted OR (95% CI)
Age*			
Age < 45	130706 (73.47)	Ref	Ref
Age ≥ 45	9570 (72.51)	0.95 (0.92,0.99)	0.96 (0.92, 0.99)
Overweight/Obeset			
No	98174 (70.95)	Ref	Ref
Yes	42102 (79.84)	1.62 (1.58, 1.66)	1.66 (1.62, 1.70)
Hypertensiont			
No	117860 (72.40)	Ref	Ref
Yes	22416 (79.15)	1.45 (1.40, 1.49)	1.44 (1.40, 1.49)
Hypercholesterolemia†			
No	112343 (71.74)	Ref	Ref
Yes	27933 (80.93)	1.67 (1.62,1.72)	1.68 (1.63, 1.73)
Vascular Diseaset			
No	139210 (73.37)	Ref	Ref
Yes	1066 (77.30)	1.24 (1.10, 1.40)	1.23 (1.08, 1.39)

PCOS, Polycystic Ovary Syndrome; CI, Confidence Interval

* Model only adjusted for time in study

† Model adjusted for time in study and age at PCOS diagnosis

## Data Availability

The data that support the findings of this study are available from Merative Truven MarketScan but restrictions apply to the availability of these data, which were used under license for the current study, and so are not publicly available. Data are available from the vendor: https://marketscan.truvenhealth.com/marketscanportal/#

## Abbreviations

PCOS: Polycystic Ovary Syndrome
ACOG: American College of Obstetricians and Gynecologists
OGTT: Oral Glucose Tolerance Test
A1C: Hemoglobin A1C
ICD: International Classification of Diseases
CPT: Current Procedural Terminology
AES: Androgen Excess Society
ADA: American Diabetes Association
OR: Odds ratio
CI: Confidence interval
